# Assessment of left ventricular performance in heart transplant recipients by three-dimensional speckle tracking imaging

**DOI:** 10.1097/MD.0000000000008129

**Published:** 2017-10-13

**Authors:** Dan Wang, Li Zhang, Qingyu Zeng, Mingxing Xie

**Affiliations:** a Department of Ultrasound, Union Hospital, Tongji Medical College, Huazhong University of Science and Technology; b Hubei Province Key Lab of Molecular Imaging, Wuhan, China.

**Keywords:** echocardiography, heart transplant, left ventricular function, three-dimensional speckle tracking imaging

## Abstract

To calculate left ventricular (LV) global performance values in heart transplant (HT) recipients by three-dimensional speckle tracking imaging (3D-STI) and to observe the changes in LV global performance over time after HT and investigate the correlated factors.

The 30 HT patients were divided into 2 groups according to postoperative time: 1 month postoperatively (HT-1) group and 6 months postoperatively (HT-2) group. Thirty healthy subjects were enrolled as control group. 3D-STI was performed to assess LV torsion, LV systolic dyssynchrony index (SDI), and LV global strain (GS). Global performance index (GPI) was calculated, and correlations factors with GPI were studied.

Heart rate (HR), left atrium (LA), interventricular septum thickness (IVST), left ventricular posterior wall thickness (LVPWT), and left ventricular mass (LVM) in both HT groups were higher than those in the control group. Compared with the control group, SDI was significantly higher in both HT groups, and SDI of the HT-1 group was much higher than that of HT-2 group. Compared with the control group, apical rotation (RoA), twist and torsion in the both HT groups decreased significantly. There were no significant differences in these values between the 2 HT groups; Basal rotation (RoB) showed no significantly difference among the 3 groups. GS in the both HT groups decreased significantly compared with the control group, and there were no significant differences in these values between the 2 HT groups. GPI of the both HT groups was significantly lower than that of the control group; however, GPI of HT-2 group was higher than that of HT-1 group. Multivariate stepwise regression analysis identified global left ventricular longitudinal peak systolic strain (GLS), the time length since surgery, left ventricular mass (LVM), and RoA as predictors of LV GPI. GLS was the most influential to GPI.

The values of LV rotation, twist and SDI can be used to assess the LV systolic function and dyssynchrony. The GPI value based on 3D-STI may accurately reflect LV performance changes over time after HT. The GPI value has potential applications in clinical practice. GLS, the time length since surgery, LVM and RoA values can be the predictors of LV global performance, and as long as the left ventricular ejection fraction (LVEF) is preserved, the left ventricular global performance of HT recipients remains stable, and tends to improve over time after HT.

## 1 Introduction

Heart transplant (HT) patients had a series of injury suffered from the operation, such as ischemia reperfusion, denervation, ischemic time before transplantation, long term use of immunosuppressive agents, and many other factors. After heart transplantation, to adapt to the postoperative environment, the heart must go through a series of adaptations to meet the needs of various physiological conditions.^[1,2]^ Thus, we may assume the left ventricular (LV) global performance was altered in patients treated with HT. Moreover, it is important to identify the early LV dysfunction of such HT patients who are clinically stable, which may provide a reference for clinical diagnosis and therapy.

It has been found that HT patients often have subnormal functional capacity, even in the absence of overt LV systolic failure or significant allograft vasculopathy.^[1,2]^ Although previous investigations have addressed myocardial mechanics after heart transplantation, focusing either on the diagnosis of rejection or allograft vasculopathy, they have all used tissue Doppler imaging (TDI) or two-dimensional speckle tracking imaging (2D-STI) technology. However, TDI-derived parameters are angle-dependent and have frame-rate limitations.^[3,4]^ 2D-STI has been developed as an angle-independent echocardiographic modality to evaluate cardiac mechanical function.^[5]^ More recently, the three-dimensional speckle tracking imaging (3D-STI) has shown the potential advantage to overcome the limitations of 2D-STI for the assessment of LV global and regional systolic function.^[6,7]^ The aim of this study was to investigate the usefulness of technology of 3D-STI and the value of global performance index (GPI) in the evaluation of global LV myocardial performance. YU^[8]^ have found that GPI has a high sensitivity and specificity in the evaluation of the cardiac toxicity of chemotherapy drugs in children and is helpful for the early detection of hidden cardiac muscle damage.^[9]^ GPI is a reflection of the comprehensive index of LV spatial movement. It can reflect the overall LV longitudinal, radial, circumferential motion, and rotational motion. GPI also includes the coordination of the sequence of cardiac synchrony.

## 2 Materials and methods

### 2.1 Study population subjects

The study was approved by the institutional research ethics committee at Union Hospital, Tongji Medical College, Huazhong University of Science and Technology, China. All data used were anonymized. The subjects gave written informed consent. All procedures and data analysis were performed by the authors; specific contributions of all enlisted authors are provided.

A total of 30 HT patients who underwent surgery were prospectively enrolled at Union Hospital in Wuhan, China, between November 2013 and June 2014, including 26 males and 4 females with a mean age of 56.93 ± 10.01 years. We followed the 30 patients and recorded the time since surgery, which was the basis for the creation of the 2 subgroups: 1 month postoperatively (HT-1 group) and 6 months postoperatively (HT-2 group). All patients were clinically stable at the time of enrolment. Exclusion criteria included: histologic evidence of acute rejection, reduced left ventricular ejection fraction (LVEF) <55%, significant coronary vasculopathy (epicardial coronary narrowing > 50% assessed by coronary angiography), significant valvular disease, and major cardiac events since baseline examination. We followed 30 patients along with the time after surgery.

The control group consisted of 30 age-matched healthy volunteers, including 25 males and 5 females with a mean age of 57.71 ± 10.10 years. They had no history of cardiovascular disease with sinus rhythm. Other organic diseases were excluded by physical examination, biochemical tests, electrocardiograph, and echocardiography.

Demographic variables, including age and sex, were collected at the time the echocardiographic studies were performed. Informed consent was obtained. Weight, height, and blood pressure measurements were also obtained in all subjects.

When the regression analysis of GPI was performed, we also had another 62 heart transplant patients who had the surgery between December 2013 and February 2015. We got the value of global left ventricular longitudinal peak systolic strain (GLS), global left ventricular circumferential peak systolic strain (GCS), apical rotation (RoA), basal rotation (RoB), and twist and left ventricular mass (LVM) of each patient.

### 2.2 Standard echocardiographic image acquisition examination

All subjects underwent complete transthoracic echocardiography (TTE) to determine cardiac structure, chamber size, and cardiac function according to the recommendations of the American Society of Echocardiography (ASE). A commercially available system (iE33; Philips Medical Systems, Andover, MA) was used with S5–1 broadband phased-array transducers. Subsequently, all subjects underwent 3D echocardiography using the same system with an X5–1 transducer. Images were optimized to obtain the entire left ventricle in a full-volume apical 4-chamber view. Data sets were acquired using a 7-heart beat acquisition setting during a period of stable heart rate, defined as the heart rate not changing during the acquisition time and not varying by >2 beats/minute during the 30 seceonds immediately before acquisition. A minimum of 4 data sets were acquired for each subject, and the 3 highest quality data sets were selected for offline analysis. Data sets that missed a portion of the LV had indistinct endocardial borders or had stitch artifacts were excluded. Image acquisition was performed by the same investigator (DW) within a 30-minute time frame.

### 2.3 Echocardiographic parameters

Cardiac chamber size includes heart rate (HR), left atrium (LA), interventricular septum thickness (IVST), left ventricular diastolic diameter (LVEDD), left ventricular posterior wall thickness (LVPWT), and LVM.

### 2.4 3D-STI

Offline 3D echocardiographic datasets analysis was performed using echocardiographic quantification (4D LV-analysis 3.0; TomTec Imaging systems, Unterschleissheim, Germany). Measurements were made using the data set with the best image quality, which was selected by consensus of the 2 readers. The frame rate of the volumetric image was 15 to 24 frames/second.

The following parameters of LV values analysis were evaluated: RoA, RoB, twist, torsion, SDI, GLS, global left ventricular peak systolic strain (GS), and GPI.

By convention, counterclockwise LV rotation as viewed from the apex was expressed as a positive value, whereas a clockwise rotation was denoted as a negative value. LV twist was defined as the net difference between the basal and apical rotation angles 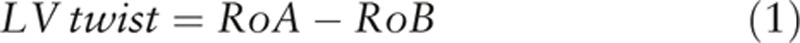


LV torsion was calculated as the net LV twist normalized with respect to ventricular end diastolic (ED) longitudinal length between the LV apex and the mitral plane 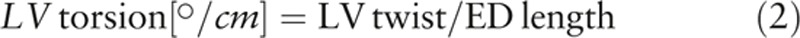


GLS was averaged over the 16 segments. The SDI was calculated as the standard deviation of the 16 times to the highest peak segmental systolic 3D strain, as a percentage of the RR interval.^[9]^ The LV GPI, which takes into account the different aspects of LV mechanics, was calculated as 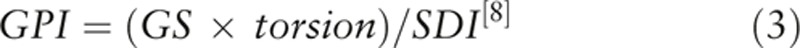


The coordinates for rotation, twist, and torsion for each frame were exported to a spreadsheet (Excel 2015; Microsoft Corporation, Redmond, WA).

### 2.5 Statistical analysis

All demographic, conventional echocardiographic, and 3D data are presented as numbers or percentages (mean ± SD). One-way analysis of variance was used to compare the data of the 3 groups. Multiple linear regression analysis was performed to determine the additional effect of the GPI of HT patients. *P* < .05 were considered statistically significant. Statistical analyses were performed using SPSS for Windows version 17.0 (SPSS, Inc., Chicago, IL).

## 3 Results

### 3.1 Clinical and echocardiographic characteristics of the population

There were no significant differences among the 3 groups in terms of age, height, weight, body surface area or LVEDD (*P* > .05). However, HR, LA, IVST, LVPWT, and LVM in both HT groups were higher than those in the control group (*P* < .05); and there were no significantly differences between the 2 HT groups in terms of HR, LA, IVSD, or LVPWT (*P* > .05) (Table 1).

**Table 1:**
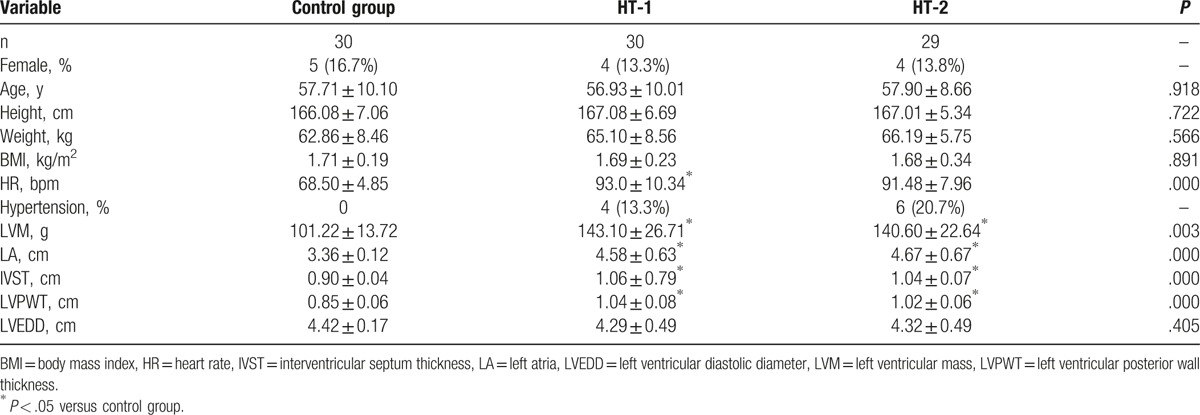
General information and conventional echocardiographic measurements among 3 groups.

### 3.2 Left ventricular synchrony findings

**Figure 1:**
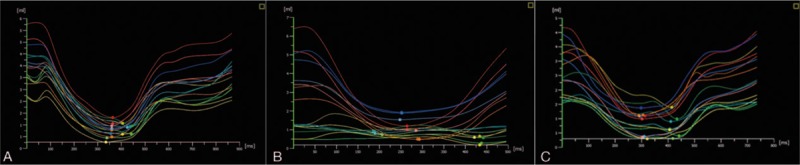
The analysis of left ventricular 16-segment end systolic volume-time curve. A, the control; (B) HT-1: 1 month postoperatively; (C) HT-2: 6 months postoperatively. The left ventricular 16-segment volume-time curves of HT-1 and HT-2 are messy and have a poor consistency. The trough of the 16-segment curves is discrete, especially in the HT-1 group. The SDI of HT increases significantly compared with the normal groups. Compared with the control group, SDI was significantly higher in both HT groups; and SDI in the HT-1 group was much higher than in the HT-2 group.

The left ventricular 16-segment volume-time curves of HT-1 and HT-2 are messy and have a poor consistency. The trough of the 16-segment curves is discrete, especially in the the HT-1 group. The SDI of HT increases significantly compared with the normal groups. Compared with the control group, SDI was significantly higher in both HT groups (*P* < .05); and SDI in the HT-1 group was much higher than in the HT-2 group (*P* < .05) (Fig. 1).

### 3.3 LV twist function

**Figure 2:**
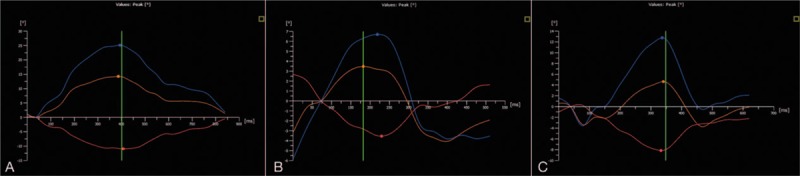
The analysis of LV rotation at apical (orange line), rotation at basal (red line) and twist (blue line) variables can be derived and displayed. A, the control; (B) HT-1: 1 month postoperatively; (C) HT-2: 6 months postoperatively. Compared with the control group, RoA, and twist in the both HT groups decreased significantly; there were no significant differences in these values between the 2 HT groups, and RoB showed no significantly difference among the 3 groups.

Compared with the control group, RoA and twist in the both HT groups decreased significantly (*P* < .05); there were no significant differences in these values between the 2 HT groups, and RoB showed no significantly difference among the three groups (*P* > .05) (Fig. 2) (Table 2).

**Table 2:**
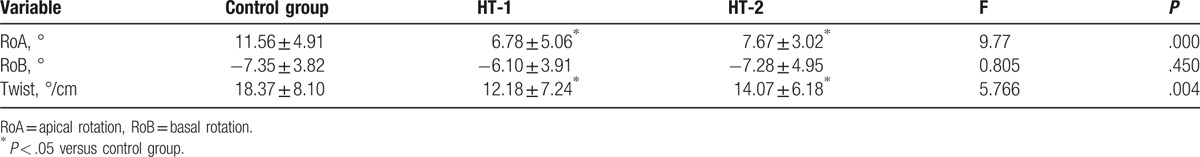
Left ventricular twist and rotation of study subjects among three groups.

### 3.4 GPI comparisons and regression analyses

**Figure 3:**
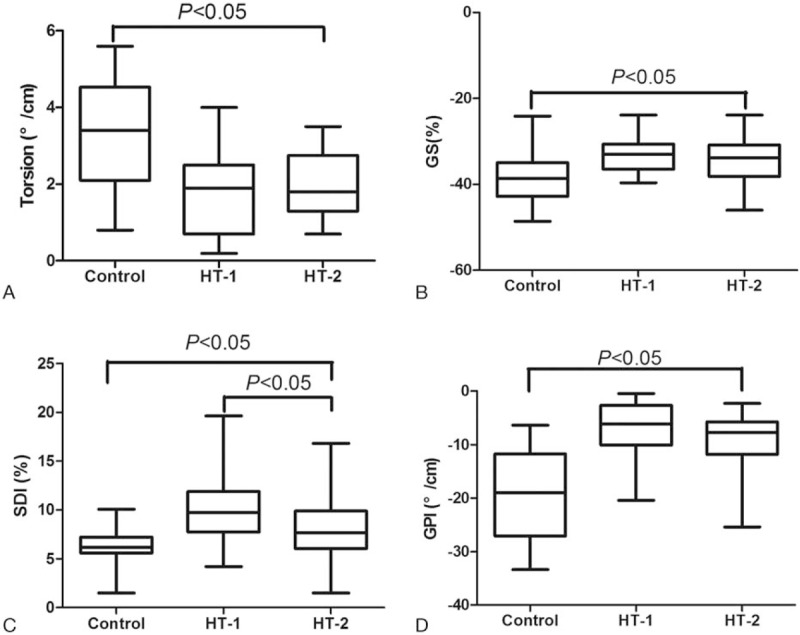
Comparison of LV torsion, GS, SDI, and GPI parameters among 3 groups. A, compared with the control group, torsion in the both HT groups decreased significantly; there were no significant differences in these values between the 2 HT groups. B, compared with the control group, GS in the both HT groups decreased significantly; there were no significant differences in these values between the 2 HT groups. C, compared with the control group, SDI was significantly higher in both HT groups; and SDI in the HT-1 group was much higher than in the HT-2 group. D, GPI in the both HT groups was significantly lower than that in the control group, GPI in HT-2 group was higher than that in the HT-1 group.

Compared with the control group, Torsion in the both HT groups decreased significantly (*P* < .05); there were no significant differences in these values between the 2 HT groups (*P* > .05). Compared with the control group, GS in the both HT groups decreased significantly (*P* < .05); there were no significant differences in these values between the 2 HT groups (*P* > .05). Compared with the control group, SDI was significantly higher in both HT groups (*P* < .05), and SDI in the HT-1 group was much higher than in the HT-2 group (*P* < .05). GPI in the both HT groups was significantly lower than that in the control group (*P* < .05); however, GPI in HT-2 group was higher than that in the HT-1 group (*P* > .05) (Fig. 3) (Table 3).

**Table 3:**
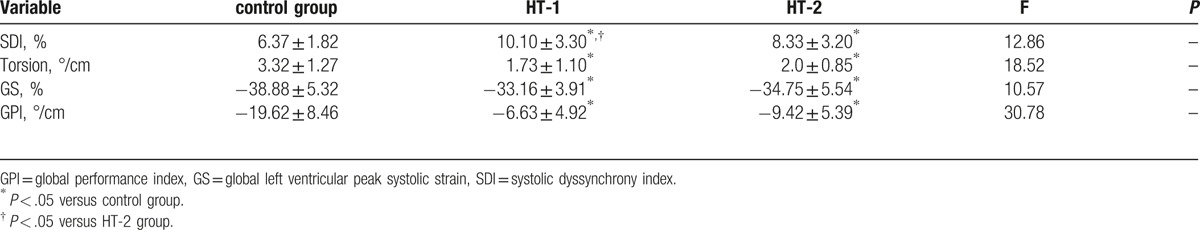
GPI related calculations.

Multivariate stepwise regression analysis identified GLS, the time since surgery, LVM, and RoA as predictors of LV GPI; of these, GLS was the most influential to GPI. As a result, the following equation was derived: 
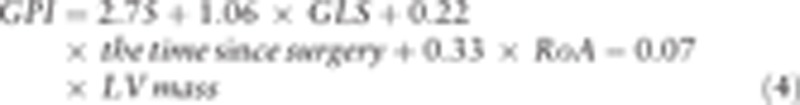


The equation shows that GLS exerts the greatest influence on the GPI value (Table 4).

**Table 4:**
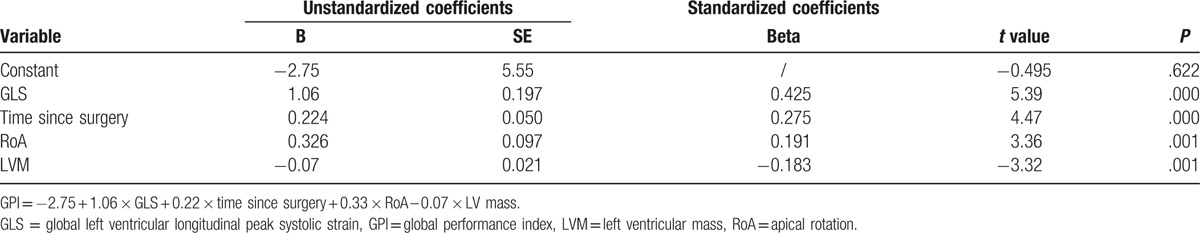
Regression analysis model of the factor of LV GPI.

## 4 Discussion

Early invasive examinations and many other research have demonstrated that LV function of the HT patients with obvious rejection is impaired,^[10,11]^ although others with a relatively stable clinical response and no obvious rejection will undergo a series of changes to meet the needs of the body and the changes of environment. The 3D-STI depends on the ability of the specking to track the movement of the heart muscle more accurately and objectively in the 3D space. 3D-STI can not only track the longitudinal, radial, and circumferential motion, but also the twist and rotational motion of the LV. Therefore, it can demonstrate the global systolic function of the LV more comprehensively.^[12,13]^

### 4.1 The typical regular changes of HT patients

Our research indicates that the HR of HT patients becomes higher than in healthy individuals. This change may be related to the denervation of the transplanted heart. HR is entirely reliant on the independently electrical activity of the sinoatrial node. The HR of healthy individuals is not only regulated by sinoatrial node, but is also controlled by the vagus and sympathetic nerves. The vagus nerve is mainly responsible for the negative regulation of the heart rate. Its effect is higher than the positive heart rate effect of sympathetic nerves. The denervation of the transplanted heart impairs the vagal response and therefore causes a significant increase in resting heart rate. In our research, the highest heart rate is as high as 94 to 106 beats/minute.

Our research shows that the LA, IVST, LVPWT, and LVM of a HT patient are obviously higher than those of healthy individuals. The cases in our research all represent are all orthotopic heart transplantation, in which the left atrial posterior wall remains and coincides with the structure of the donor heart. During the operation, the reconstruction of a double atrium, namely, the separation of atrial and electrical activity, will all cause the expansion of double atria after surgery. Meanwhile, the operation itself and ischemia reperfusion will all cause edema of the myocardial cells. These changes caused by surgery will resolve or disappear with the passage of time. It is also important to note that the adaptive changes of the transplanted heart and long-term use of immunosuppressive agents could also cause the reconstruction of the transplanted heart, and cause an obvious increase in IVST, LVPWT, and LVM. These effects are considered adaptive changes of the transplanted heart.

### 4.2 Evaluating the changes of LV strain of HT patients

Denervation is the most basic pathophysiological characteristics of the HT patients. Hemodynamics, endocrine, electrical physiology, responses to external stimuli, and pharmacological reactions of a transplanted heart are different from the normal heart when it has lost neural control. After denervation, the peripheral vascular resistance of a HT patient decreases. Central venous pressure soon decreases accordingly. The regulating axis among renin, angiotensin and aldosterone will be damaged, which might cause capacity overload, namely, the increase in preload of the heart. After denervation of the outgoing nerve, the capacity of catecholamines in transplanted cardiac muscle is quickly depleted. However, the contractile function of the transplanted heart was completely dependent on the end diastolic ventricular volume and the catecholamine in blood circulation, which maintain a normal increase in cardiac output.^[8]^ The influence of the effect of central nervous system vascular signals on cardiovascular system disappears, and the low blood volume and the mechanism of blood vessel relaxation activation of adrenal signal will be affected.^[10]^

Our research shows that there is no significant difference in LVEF between HT patients and healthy individuals. The strain of the entire left ventricle is significantly lower than in normal hearts. K Haydar et al^[14,15]^ reported research after 40 patients who had undergone the HT operations for 1 year. Tissue velocity imaging showed that the LV response was significantly lower than that of the normal heart. This finding is consistent with the results of our research and revealed that the LV systolic function of the transplanted heart had been obviously impaired, possibly through a series of injuries the patient might have suffered during the operation, such as ischemia reperfusion, cell rejection reactions after surgery, and long-term use of immunosuppressive agents. Specifically, ischemia reperfusion could lead to myocardial infarction and apoptosis. The aforementioned factors will all cause the decreasing of the LV strain of the transplanted heart.

### 4.3 Evaluating the changes of LV synchrony of HT patients

The effective pumping blood of the normal human heart is mainly relying on the rhythmically synchronized contraction and relaxation of the ventricular wall. The LV systolic and diastolic function can be affected by the decreases in the ventricular wall motion, which will cause changes in the hemodynamics. Other researches have shown that there are advantages in quantitatively evaluating the LV function and the coordination and synchronization of LV myocardial contraction of a HT using 3D-STI. SDI can be used as an effective index for evaluating LV systolic synchrony in HT patients.^[16]^

Our research shows that the left ventricular 16-segment volume-time curve is smoothing and makes corresponding curves. The time when the curve reaches the LV end systolic volume (curve trough), that is, the trough of the 16-segment volume-time curve is more concentrated. The LV 16-segment volume- time curves of 1-months and 6-months postsurgery groups are messy and have poor consistency. The troughs of the 16-segment curves are discrete, especially in the 1-months postoperative group. The SDI of the HT increases significantly compared with the normal groups. Because the recipient heart is often significantly expanded after implantation of the transplanted heart to the location where the recipient heart had been removed, the implanted heart may require a location change.^[17]^ Even the mass activity of the implanted heart will increase of swing, which will also cause a decrease of synchronism. Atrial anastomosis maintains the sinoatrial nodes of the recipient heart. This condition will cause the transplanted heart to have 2 sinoatrial nodes at the same time. This separation of the atrial electrical activity and the contrast in electric activities will cause nonsynchronous mechanical activity to varying degrees. The synchrony of LA contraction will cause a decrease in the coordination and synchronization of LV wall passive relaxation. The SDI of the 6-months postoperative group was smaller than that of the 1-months postoperative group. This difference indicated that the LV synchrony of HT patients 6 months after surgery becomes better than that of patients 1 month after surgery. This difference reinforces that the operation itself and ischemia reperfusion also influence the synchronization of transplanted hearts. With the prolongation of postoperative time, the synchronization of the myocardium will improve because of the partial recovery of LV myocardial from damage.

### 4.4 Evaluating the changes in the LV torsion and rotational motion of HT patients

The spatial movement of the heart is very complex. Ventricular wall motion mainly includes inward contraction of the myocardium, with rotation of the heart along the axis and the level of the heart and stretching, twisting, and other local movement.^[18]^ Larger spatial location changes will occur when the transplanted heart has been implanted in the body and its activity increases significantly. These factors will all influence the torsion and rotation of the heart itself.

The LV myocardium is a myocardial band which is formed in a spiral fashion. Contraction and relaxation of the left ventricle result in the LV systolic torsion similar to twisting a towel and the diastolic untwisting.^[19]^ Therefore, we can evaluate the LV systolic and diastolic function base on LV torsion.^[20]^ The torsional motion of the heart refers to the rotational motion around the LV short axis center as the imaginary circle. From the apex to the bottom of the heart, we can observe that the apex rotates counterclockwise when contracting; however, the bottom of the heart rotates clockwise. This action forms the torsional motion of the left ventricle along the long axis. This type of torsional motion plays an important role in the LV systolic and diastolic function.^[21]^ Any pathological condition, such as aortic stenosis and coronary artery disease, may affect LV torsion and rotational motion, such as aortic stenosis and coronary artery disease.^[22,23]^ The direction and size of the LV torsion depend on the cross-wall stress of the LV myocardium and the mechanical advantages of myocardial fibers of the epicardium compared with that of the endocardium.^[24]^ Research shows that the myocardial torsion angle and the cross-wall stress gradually decrease from the apex to the base at the time of the contraction. Our research demonstrates that the LV apical rotation and LV torsion of HT patient are significantly lower than those in healthy individuals. Because the LV torsion is very sensitive to sympathetic nerve stimulation, the denervation of the transplanted heart will seriously influence the LV torsion.^[25,26]^ Ischemia, hypoxia, and the transplant procedure will all cause different degrees of injury to the myocardium, resulting in damage to the LV twist and rotation function.^[27]^ After HT, caused by a variety of factors including ischemia reperfusion injury, allograft vasculopathy immune injury and long-term immunosuppression, the left ventricle will be remodeled, the myocardium of the left ventricle will be damaged and the function of LV torsion will deteriorate. The decrease in LV torsion and rotation in HT patients may be related to the fact that the transplanted heart has no limitation of pericardium because the pericardium plays a supporting and maintaining role in the twisting and rotating motion of the heart. Our research shows that the LV apical rotation of HT patients is significantly lower than in healthy individuals; there is no significant difference between normal and transplanted hearts regarding the LV rotation angle of the basal part. For normal hearts, the LV apex is relatively free. Its range of motion increases significantly compared with the basal part of the left ventricle. The LV apex is dominant in the torsional and systolic function of the left ventricle; thus, the decrease in LV apical torsion is more obvious than that of the basal part.

### 4.5 Evaluating the LV GPI of HT patients

Our research shows that the LVM of the transplanted heart is significantly higher than in healthy individuals. Detecting abnormal changes in the LV mechanics of HT patients would provide a useful reference for clinical determination of the damage of LV systolic function. With greater postoperative time, the ischemic reperfusion injury suffered in surgery, the cell rejection reaction after surgery, and the long-term use of immunosuppressive agents and any other factors will all interact. There will be some changes in the LV strain, torsion rotation and synchrony of the transplanted heart. Our research analyzes the LV strain, introduces the LV twist and synchrony and measure the GPI based on 3D-STI. It shows that the LV GPI of HT patients is lower than that in healthy individuals. This change is related to decreases in LV strain, rotation, torsion, and synchrony. Furthermore, the GPI of 6 months after surgery shows an increasing trend compared with the 1 month after surgery, although the difference is not statistically significant, which may be related to the short postoperative time of the patients we included and the small number of patients. Our research shows that the LV GPI of HT patients is lower than that in healthy individuals. This difference can more comprehensively reflect the different degrees of damage to LV function in HT patients whose LVEF value is “normal.” Furthermore, we used multiple linear stepwise regression in our study to find the factors that may affect the overall function index of the left ventricle. Multivariate stepwise regression analysis identified GLS, the time length since surgery, LVM, and RoA as predictors of LV GPI. GLS was the most influential to GPI. At the same time, this finding also indicates that the overall function of the left ventricle gradually improves with time after surgery, possibly related to resolution of edema myocardial cells. Early temporarily impaired cardiac function can be gradually restored. With the extension of partial nerve regeneration after surgery, the related functions of neural regulation can be partially recovered.

The main patients in our research are relatively stable clinical heart transplant patients whose LVEF values are in normal ranges and who have no obvious signs of rejection. GPI parameters overall reflect the function of the left ventricle in these patients in terms of left ventricular strain, torsion and synchronization. This parameter can provide a reference for clinical diagnosis. The left ventricular global performance of HT recipients remains stable and tends to improve over time after HT, which can be a certain basis for the future monitoring of transplant rejection, coronary artery disease and other serious complications

### 4.6 Limitations

Limitations of our research include the follow-up time that was too short to reflect the change of the function of HT patients after surgery. The frame rate SD-STI was low, which could influence on speckle tracking ability. Furthermore, the number of patients in our research was too small. Future studies assessing left ventricular performance in HT over time after surgery is needed as it looks promising.
